# HSP90 and Aha1 modulate microRNA maturation through promoting the folding of Dicer1

**DOI:** 10.1093/nar/gkac528

**Published:** 2022-06-23

**Authors:** Xiaochuan Liu, Yen-Yu Yang, Yinsheng Wang

**Affiliations:** Department of Chemistry, University of California, Riverside, Riverside, CA 92502, USA; Department of Chemistry, University of California, Riverside, Riverside, CA 92502, USA; Department of Chemistry, University of California, Riverside, Riverside, CA 92502, USA

## Abstract

Aha1 is a co-chaperone of heat shock protein 90 (HSP90), and it stimulates the ATPase activity of HSP90 to promote the folding of its client proteins. By employing ascorbate peroxidase (APEX)-based proximity labeling and proteomic analysis, we identified over 30 proteins exhibiting diminished abundances in the proximity proteome of HSP90 in HEK293T cells upon genetic depletion of Aha1. Dicer1 is a top-ranked protein, and we confirmed its interactions with HSP90 and Aha1 by immunoprecipitation followed by western blot analysis. Genetic depletion of Aha1 and pharmacological inhibition of HSP90 both led to reduced levels of Dicer1 protein. Additionally, HSP90 and Aha1 bind preferentially to newly translated Dicer1. Reconstitution of Aha1-depleted cells with wild-type Aha1 substantially rescued Dicer1 protein level, and a lower level of restoration was observed for complementation with the HSP90-binding-defective Aha1-E67K, whereas an Aha1 mutant lacking the first 20 amino acids—which abolishes its chaperone activity—failed to rescue Dicer1 protein level. Moreover, knockdown of Aha1 and inhibition of HSP90 led to diminished levels of mature microRNAs (miRNAs), but not their corresponding primary miRNAs. Together, we uncovered a novel mechanism of HSP90 and Aha1 in regulating the miRNA pathway through promoting the folding of Dicer1 protein, and we also demonstrated that Aha1 modulates this process by acting as an autonomous chaperone and a co-chaperone for HSP90.

## INTRODUCTION

Heat shock protein 90 (HSP90) is a key regulator of proteostasis in cells under both physiological and stress conditions. Several hundred client proteins have been identified for HSP90, rendering HSP90 a central modulator for many important biological processes, including protein folding, development, DNA repair, immune response, etc. (1–5). Co-chaperones are important regulators of HSP90, where they modulate the HSP90 chaperone cycle or act as adaptor proteins to promote client engagement (6,7). Depending on the presence of tetratricopeptide repeat (TPR) domain, HSP90 co-chaperones can be divided into TPR-containing (e.g. FKBP51, FKBP52, HOP, CNS1 and PP5) and non-TPR-containing (e.g. P23, CDC37 and Aha1) groups (8).

A large number of co-chaperones interact with HSP90 to regulate the ATPase-associated conformational changes of the HSP90 dimer that occur during client processing. Among the co-chaperones, CDC37 interacts with a subset of client proteins of HSP90 and acts as the major partner of HSP90 in assisting the folding of protein kinases (9–11). In addition, FKBP51 and FKBP52 co-chaperones promote the folding and maturation of steroid hormone receptors (12).

Aha1 is a stress-regulated co-chaperone of HSP90, where it potently stimulates the ATPase activity of HSP90 by promoting it to adopt the N-terminally dimerized, closed state; this process involves the asymmetric binding of Aha1 to the middle and the ATP-binding N-terminal domains of HSP90 (13–15). Apart from this role in stimulating the ATPase activity of HSP90, Aha1 also functions in late stage of HSP90 chaperone cycle (13–15). In addition, SUMOylation of lysine 191 in HSP90 promotes the recruitment of Aha1 (16). On the other hand, Aha1 was found to enhance the misfolding of cystic fibrosis transmembrane conductance regulator and the disease-relevant Δ508 mutant found in cystic fibrosis patients by diminishing the dwell time of these difficult-to-fold client proteins on HSP90 (17). Moreover, Aha1 promotes production of pathological tau aggregates (18). Nevertheless, other client proteins specifically regulated by Aha1 remain largely undefined. Hence, the identification of client proteins of the HSP90–Aha1 network will shed light on Aha1’s role in HSP90-regulated biological processes and provide new insights into the implications of Aha1 in human diseases.

Proximity labeling together with proteomic analysis allows for the identification of proteins that interact weakly and/or transiently with a target protein of interest in the native cellular environment, and the method is invaluable in profiling functional components of various protein complexes (19). In proximity labeling, an enzyme, capable of catalyzing the biotinylation of endogenous proteins in a proximity-dependent manner, is fused with the target protein of interest, which enables proximal and interacting proteins to be tagged with biotin for subsequent affinity enrichment and mass spectrometric analysis (19–21).

Currently, proximity labeling methods are based on the use of a mutant *Escherichia coli* biotin ligase BirA^R118G^ (BioID) (20,21) or an engineered ascorbate peroxidase (APEX) (22,23). BioID relies on the generation of an activated biotin adenylate ester (biotin-AMP) in the BirA active site, and the released biotin-AMP reacts with lysine side chains of nearby proteins in cells (21). Although BioID facilitates the identification of proteins exhibiting weak and/or transient interactions with the target protein of interest, the slow reaction kinetics entails a relatively long labeling time to attain appreciable levels of biotinylation (24,25). This hampers the study of short-lived states of a cell or organelle’s rapid responses to intracellular cues and extracellular stimuli. APEX is an engineered peroxidase that functions as a promiscuous labeling enzyme for live-cell proteomics. Upon addition of hydrogen peroxide to cells preloaded with a biotin phenol substrate, APEX generates biotin-phenoxyl radicals, which exhibit a half-life of <1 ms and react with amino acid residues in proximal endogenous proteins (22,23). Since the limited sensitivity of APEX hampers its applications involving low level of APEX expression, APEX2 with improved catalytic efficiency has been developed and it enables superior enrichment of endogenous proteins (23).

Here, we employed APEX2 labeling, in combination with label-free quantification, for the identification of client proteins of co-chaperone Aha1. We established Dicer1 as a new client protein of Aha1 and demonstrated the importance of HSP90 and Aha1 in modulating microRNA (miRNA) maturation.

## MATERIALS AND METHODS

### Materials

All chemicals, unless otherwise stated, were purchased from Sigma-Aldrich. Cycloheximide (CHX), 17-dimethylaminoethylamino-17-demethoxygeldanamycin (17-DMAG), AT13387 and ganetespib were purchased from Fisher Scientific.

### Cell culture

HEK293T, U2OS and GM00637 cells were obtained from ATCC. These cells were maintained in Dulbecco’s modified Eagle medium (DMEM) supplemented with 10% fetal bovine serum (Invitrogen) and 1% penicillin/streptomycin (at 10 000 U/ml, Thermo Fisher). The cells were cultured at 37°C in a humidified atmosphere containing 5% CO_2_.

### shRNA and plasmids

All oligodeoxyribonucleotides used for the construction of small-hairpin RNA (shRNA) plasmids were obtained from Integrated DNA Technologies (sequences are listed in Supplementary Table S2). Control shRNA with a hairpin sequence of 5′-CCT AAG GTT AAG TCG CCC TCG CTC TAG CGA GGG CGA CTT AAC CTT AGG-3′ (Addgene, Cambridge, MA, USA) was employed as a negative control, as described previously (26). All shRNAs were cloned into the AgeI and EcoRI sites of the pLKO.1 vector (Addgene, plasmid #10878). The coding sequence of HSP90α was cloned into the NheI and NotI restriction sites of the V5-Apex2 vector (Addgene, plasmid #72480) to yield the HSP90–Apex plasmid, where APEX was fused to the C-terminus of HSP90α protein. The coding sequences of wild-type Aha1, Aha1-E67K and Aha1-Δ20, where the first 20 amino acids were deleted, were cloned into the BamHI and EcoRI sites of the pRK7 3× Flag vector (Addgene, plasmid #8996). pCAGGS-Flag-hsDicer1 was purchased from Addgene (#41584). All constructs were confirmed by Sanger sequencing.

### Lentivirus production and stable cell line generation

HEK293T cells were transfected with pLKO.1/puro-shRNA plasmids together with pLTR-G (plasmid #17532) envelope plasmid and pCMV-dR8.2 dvpr (plasmid #8455) package plasmid using PolyFect Transfection Reagent (Qiagen). Viral particles were collected 48 h later and filtered through a 0.45-μm sterile filter. The cells were infected with a 5:1 mixture of viral particle solution and DMEM for 48 h, screened with 1 μg/ml puromycin for a week and cultured in complete DMEM supplemented with the same concentration of puromycin.

### Proteomic sample preparation and LC–MS/MS analysis

Proximity labeling was conducted as described previously (23). In brief, ∼2 × 10^7^ cells were incubated in complete medium containing 500 μM biotin phenol for 30 min, followed by incubation with 1.0 mM H_2_O_2_ for 1 min. The reaction was terminated by washing the cells with a buffer containing 5 mM 6-hydroxy-2,5,7,8-tetramethylchroman-2-carboxylic acid (Trolox), 10 mM sodium ascorbate and 10 mM sodium azide in phosphate-buffered saline (PBS) and PBS twice each. The cells were lysed with 500 μl CelLytic M (Sigma) containing 1% protease inhibitor cocktail (Sigma) and the above-described quenching buffer. The cell lysates were centrifuged at 13 000 rpm at 4°C for 10 min to remove the cell debris.

Approximately 1 mg of total proteins were incubated with streptavidin-conjugated agarose beads (Pierce Thermo) at 4°C overnight. The beads were subsequently washed with 500 μl of CelLytic M twice, 1 M KCl and 0.1 M Na_2_CO_3_ once each, and again with CelLytic M twice. Biotinylated proteins were eluted by incubating the beads in 3× SDS-PAGE loading buffer containing 2 mM biotin at 95°C for 5 min. The resulting proteins were resolved on a 15% SDS-PAGE gel for ∼1 cm. Gel bands corresponding to protein molecular weight range of >15 kDa were excised and cut into 1 mm^3^ cubes for in-gel tryptic digestion (27). Cysteine reduction and alkylation were conducted by incubating the gel pieces in 10 mM dithiothreitol and 55 mM iodoacetamide at 37°C for 1 h and at room temperature in darkness for 20 min, respectively. The proteins were subsequently digested with 200 ng modified MS-grade trypsin (Pierce) in 50 mM NH_4_HCO_3_ at 37°C overnight. The peptide mixture was dried in a Speed-vac and desalted with OMIX C18 pipette tips (Agilent Technologies).

The ensuing peptides were reconstituted in 0.1% formic acid, and 10% of the mixture was subjected to LC–MS/MS analysis on a Q Exactive Plus quadrupole-Orbitrap hybrid mass spectrometer (Thermo Fisher Scientific) coupled with an Ultimate 3000 UPLC system. The samples were automatically loaded onto a 3-cm trapping column (150 μm i.d.) packed with ReproSil-Pur 120 C18-AQ resin (5 μm in particle size and 120 Å in pore size, Dr. Maisch GmbH HPLC) at a flow rate of 3 μl/min. The trapping column was coupled to a 20-cm home-made fused silica analytical column (PicoTip Emitter, New Objective, 75 μm i.d.) packed with ReproSil-Pur 120 C18-AQ resin (3 μm in particle size and 120 Å in pore size, Dr. Maisch GmbH HPLC). The peptides were resolved using a 140-min linear gradient of 4.0–29.6% acetonitrile in 0.1% formic acid at a flow rate of 300 nl/min. The mass spectrometer was operated in a data-dependent acquisition mode. The spray voltage was 2.0 kV. The 25 most abundant ions detected in MS were isolated and activated in the HCD cell at a collision energy of 28 to yield the MS/MS, which were acquired in the Orbitrap analyzer at a resolution of 17 500 and with an automatic gain control target of 1 × 10^5^.

Raw LC–MS/MS data were processed using MaxQuant (version 2.0.1.0) (28). Methionine oxidation and N-terminal acetylation were specified as variable modifications, and cysteine carbamidomethylation as a fixed modification. A mass tolerance of 20 ppm was set for both MS and MS/MS. A maximum of two trypsin missed cleavages were permitted, and the peptides were filtered at 1% false discovery rate (FDR). Match between runs option was enabled, and the match time window was 0.7 min. For protein identification, peptide mass spectra were searched against a target-decoy human UniProt database (UP000005640) and filtered at 1% protein FDR. Normalized label-free quantification with a minimum ratio count of 2 was used for protein quantification. The mass spectrometry data were deposited to ProteomeXchange (PXD-028980) (29,30).

### Immunoprecipitation and western blot

The cells were lysed in CelLytic™ M Cell Lysis Reagent supplemented with a complete protease inhibitor cocktail (Sigma-Aldrich). After lysis on ice for 30 min, the samples were centrifuged at 13 000 rpm for 10 min and the supernatant was collected. One milligram of total protein was incubated with prewashed anti-Flag M2 beads at 4°C overnight. For assessing the interaction between endogenous Dicer1 and Aha1 proteins, cell lysate was precleared with Protein A/G Plus agarose beads at 4°C for 2 h, and 1 mg of the resulting protein lysate was subsequently immunoprecipitated using antibodies recognizing Dicer1 and Aha1 (4 μg) at 4°C overnight. After addition of Protein A/G agarose beads, incubation was continued for another 2 h. The resulting immune complexes were washed with ice-cold lysis buffer for five times, and the beads were boiled in 2× SDS-PAGE loading buffer to elute the captured proteins, which were subjected to western blot analysis.

Antibodies recognizing human Aha1 (Santa Cruz Biotechnology, # sc-166610), HSP90 (Santa Cruz Biotechnology, # sc-13119), Dicer1 (Proteintech, # 20567-1-AP), V5 (Proteintech, # 14440-1-AP), streptavidin (Thermo Scientific, # S911), HOP (ABclonal, # A0036), IDH1 (ABclonal, # A13245) and Flag epitope tag (Cell Signaling Technology, # 14973S) were employed as primary antibodies for western blot analysis. Goat anti-rabbit IgG (whole molecule) peroxidase antibody (Sigma, # A0545) and m-IgGκ BP-HRP (Santa Cruz Biotechnology, # sc-516102) were used as secondary antibodies. Membranes were also probed with anti-GAPDH (Santa Cruz Biotechnology, # sc-32233) and anti-tubulin (Santa Cruz Biotechnology, # sc-23948) to confirm equal protein loading.

### CRISPR/Cas9-mediated ablation of *AHSA1* gene

*AHSA1* ^−/−^ HEK293T cells were generated by genome editing with the CRISPR/Cas9 system as previously described (26), where the single-guide RNAs (sgRNAs) were designed according to a previously published method (31). The guide sequences for the production of sgRNA targeting *AHSA1* gene were inserted into the hSp-Cas9 plasmid pX330 (Addgene) at the BbsI digestion sites. After transfection and clonal isolation, successful deletion of the *AHSA1* gene in single-cell clones was screened by western blot using anti-Aha1 antibody and the deleted loci in genomic DNA were identified by Sanger sequencing. The sgRNA sequences are listed in Supplementary Table S2 and the Sanger sequencing results are shown in Supplementary Figure S3.

### Real-time quantitative PCR analysis of mature miRNA

Cells were seeded in six-well plates at a 40% confluence level and treated with the indicated compounds. Total RNA was extracted from cells using TRIzol and used for cDNA synthesis as described (32). Approximately 1 μg RNA was reverse-transcribed by employing 100 units of M-MLV reverse transcriptase (Promega), 1 unit of poly(A) polymerase (New England Biolabs) and a 5′-tagged oligo(dT)_15_ primer. After a 1-h incubation at 42°C, the reverse transcriptase was deactivated by heating at 95°C for 5 min. Real-time quantitative PCR (RT-qPCR) experiments were performed using iQ SYBR Green Supermix kit (Bio-Rad) on a Bio-Rad iCycler system, and the running conditions were 95°C for 3 min and 45 cycles of 95°C for 15 s, 55°C for 30 s and 72°C for 45 s. The comparative cycle threshold (ΔΔCt) method was used for the relative quantification of mature miRNA expression (33), with the primers being listed in Supplementary Table S3. The mature miRNA level was normalized against that of *GAPDH*.

### RT-PCR analysis of primary miRNA

cDNA was synthesized using SuperScript III reverse transcriptase, and PCR was performed using Phusion DNA polymerase as previously described (34). For pri-let-7b, pri-mir-30a, pri-mir-15a and pri-mir-100 (with amplicon sizes of 400, 388, 396 and 386 nt, respectively), PCR reactions were conducted with 29, 25, 26 and 26 cycles at annealing temperatures of 65, 61, 61 and 62°C, respectively. The sequences of primer pairs spanning the stem–loop region of each miRNA are shown in Supplementary Table S4.

## RESULTS

### APEX labeling together with proteomic analysis revealed Dicer1 as a candidate client protein of Aha1

**Figure 1.**
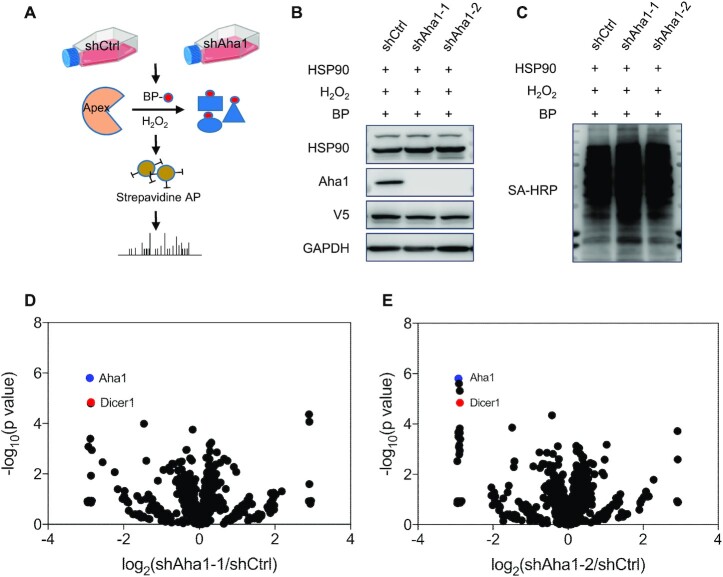
APEX labeling-based proteomic experiments revealed Dicer1 as a candidate client protein of Aha1. (**A**) A schematic diagram depicting the workflow for APEX-based proteomic analysis. ‘BP’ and ‘AP’ designate biotin phenol and affinity pulldown, respectively. (**B**) HEK293T cells with genetic depletion of Aha1 using two different sequences of shRNAs were transfected with the HSP90–APEX plasmid for 24 h, treated with biotin phenol for 30 min and H_2_O_2_ for 1 min, and the lysates were employed to monitor the expression levels of Aha1, HSP90 and V5 tag. (**C**) Western blot analysis shows the labeling efficiency of HSP90–APEX in panel (B). (**D**, **E**) Quantitative proteomic results of HEK293T cells with genetic depletion of Aha1 using two separate sequences of shRNAs compared with shControl. Dicer1 and Aha1 are marked in red and blue, respectively.

To identify potential client proteins of Aha1, we employed APEX labeling, in combination with LC–MS/MS analysis, to explore the proximity proteome of HSP90 that is perturbed by shRNA-mediated depletion of Aha1 (Figure 1A–C). To this end, we first examined whether our experimental design allows for identification of proteins whose interactions with HSP90 are known to be modulated by Aha1. Our results showed that IDH1, a known client protein of Aha1 (35), displayed lower levels of expression in HEK293T cells after Aha1 knockdown (Supplementary Figure S1). In addition, western blot analysis of HSP90–APEX-labeled samples showed that Aha1 knockdown leads to markedly diminished presence of IDH1 in the proximity proteome of HSP90 (Supplementary Figure S1). In agreement with the fact that Aha1 can displace the inhibitory co-chaperone HOP from HSP90 (6,36), our APEX labeling followed by western blot analysis revealed an augmented association of HOP with HSP90 after genetic depletion of Aha1, although the expression level of HOP was not influenced by Aha1 knockdown (Supplementary Figure S1). These results demonstrated that the HSP90–APEX fusion protein is functional in cells and proximity labeling can allow for uncovering HSP90 interaction proteins that are modulated by Aha1.

Our APEX labeling and LC–MS/MS analysis led to the identification of 31 proteins exhibiting substantial diminutions (by at least 1.5-fold) in the proximity proteome of HSP90 in HEK293T cells with stable knockdown of Aha1 with two different sequences of shRNAs versus cells treated with control, nontargeting shRNA (shControl) (Figure 1D and E, and Supplementary Table S1). Among them, Dicer1 is a top-ranked protein that can be enriched from shControl- over shAha1-treated HEK293T cells (Figure 1D and E, and Supplementary Table S1).

### Dicer1 is a client protein of Aha1

**Figure 2.**
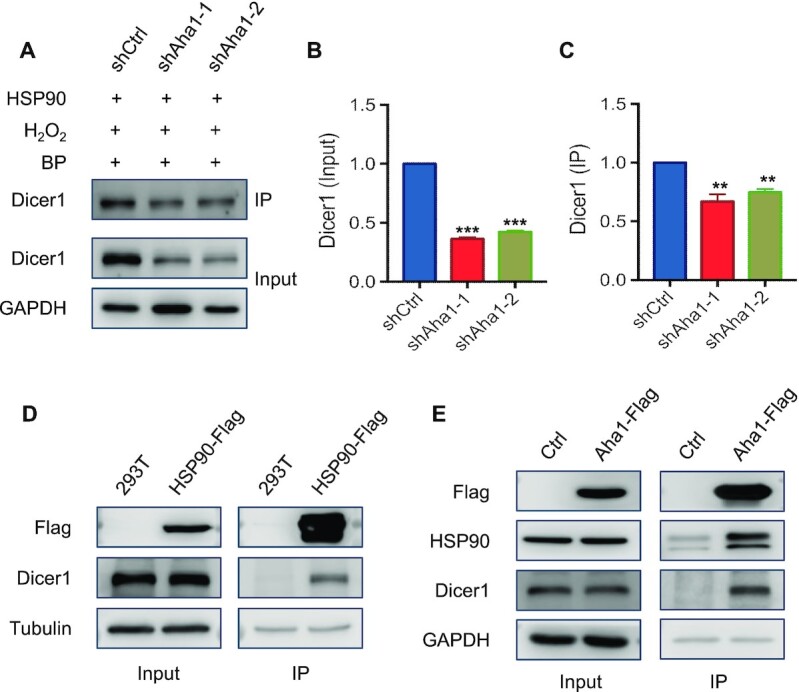
Genetic depletion of Aha1 led to diminished levels of Dicer1, and Aha1 interacts with Dicer1 in HEK293T cells. (**A**) HEK293T cells treated with shAha1 and shControl were transfected with the HSP90–APEX plasmid for 24 h and treated with biotin phenol for 30 min and H_2_O_2_ for 1 min, followed by cell lysis and streptavidin affinity pulldown, and the whole-cell lysate and pulldown lysate were used for western blot analysis. (**B**) The change in expression level of Dicer1 in the input panel of (A). (**C**) The alteration in the level of Dicer1 protein in the proximity proteome of HSP90 based on the IP panel of (A). (**D**) The lysates of HEK293T cells with 3× Flag tag on the C-terminus of endogenous HSP90 were used for Flag affinity purification, and the immunoprecipitates were employed to monitor the levels of HSP90-Flag and Dicer1 proteins by western blot. (**E**) HEK293T cells were transfected with the Flag-tagged Aha1 plasmid for 24 h, followed by cell lysis and Flag affinity pulldown, and the whole-cell lysate and pulldown lysate were used for western blot analysis. Dicer1 level in panel (B) was quantified from band intensities using ImageJ and normalized to GAPDH first and then displayed relative to the level in HEK293T shControl cells. Dicer1 level in panel (C) was quantified from band intensities using ImageJ and was displayed relative to the level in HEK293T shControl cells. The data represented the mean ± SD (*n* = 3). The *P* values were calculated using unpaired, two-tailed Student’s *t*-test: **, 0.001 ≤ *P* < 0.01; ***, *P* < 0.001.

Owing to the importance of Dicer1 in the miRNA pathway (37,38), we asked whether Dicer1 protein expression is subjected to Aha1 regulation. First, we monitored the level of Dicer1 protein in HSP90–APEX-labeled samples by using western blot analysis. Our results confirmed a decreased presence of Dicer1 in the proximity proteome of HSP90 upon genetic depletion of Aha1 (Figure 2C).

Aha1 is known to stimulate the ATPase activity of HSP90, thereby promoting the folding of its client proteins (4). We, therefore, hypothesized that Dicer1 might be a client protein of HSP90 that is modulated by Aha1. If this hypothesis is correct, we would expect to observe an attenuated level of Dicer1 protein in cells with Aha1 being depleted, and an interaction between Dicer1 and HSP90. Indeed, our western blot results showed that Dicer1 displays diminished expression level in HEK293T cells upon shRNA-mediated depletion of Aha1 (Figure 2A and B). Moreover, genetic ablation of *AHSA1* gene with CRISPR/Cas9 led to pronounced decreases in Dicer1 protein level, whereas the mRNA level of Dicer1 was not affected after shRNA- or CRISPR-mediated depletion of Aha1 in HEK293T cells (Supplementary Figures S2 and S3), suggesting that Aha1 regulates Dicer1 expression at the protein rather than the transcript level. We also found that the shRNA-mediated depletion of Aha1 led to decreased levels of Dicer1 protein in GM00637 human skin fibroblast and U2OS human osteosarcoma cells (Supplementary Figure S4).

We next explored the interaction between Dicer1 and HSP90/Aha1. Anti-Flag pulldown with the lysate of CRISPR-engineered HEK293T cells, where endogenous HSP90 is fused with a 3× Flag tag on the C-terminus (39), showed that HSP90 interacts strongly with endogenous Dicer1 (Figure 2D). Flag immunoprecipitation results also revealed a strong interaction between ectopically expressed Flag-Aha1 and endogenous Dicer1 in HEK293T cells (Figure 2E). Moreover, results from reciprocal immunoprecipitation with antibodies against Dicer1 and Aha1 proteins showed an interaction between endogenous Dicer1 and Aha1 in HEK293T cells (Supplementary Figure S5).

**Figure 3.**
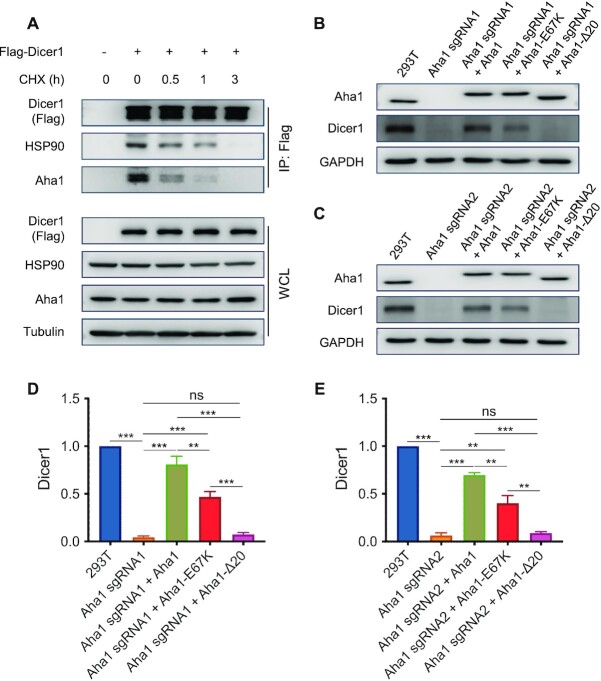
Dicer1 is a client protein of HSP90 and Aha1. (**A**) HEK293T cells were transfected with Flag-Dicer1 for 24 h, treated with CHX to block *de novo* protein synthesis and then harvested at the indicated time points. The cells were subsequently lysed and western blot was employed to monitor the changes of Dicer1 (Flag), HSP90 and Aha1 proteins, where tubulin was used as a loading control. (**B**, **C**) HEK293T cells with CRISPR-mediated ablation of Aha1 were reconstituted with wild-type Aha1, Aha1-E67K and Aha1-Δ20 for 24 h. The cells were then harvested and the ensuing lysates were used to monitor the changes in expression level of Dicer1 protein. (**D**, **E**) The data in panels (B) and (C) were quantified from band intensities using ImageJ and normalized against that of GAPDH, where the values are displayed relative to those observed in shControl cells. The data represented the mean ± SD (*n* = 3). The *P* values were calculated using unpaired, two-tailed Student’s *t*-test: ns, *P* ≥ 0.05; *, 0.01 ≤ *P* < 0.05; **, 0.001 ≤ *P* < 0.01; ***, *P* < 0.001.

Since Aha1 stimulates the ATPase activity of HSP90 to promote the folding of its client proteins, we next asked whether HSP90 and Aha1 bind preferentially to newly synthesized Dicer1. Maturation-dependent interaction assay (40) by blocking *de novo* protein synthesis with CHX revealed a progressive decline in binding of Aha1 and HSP90 with newly synthesized Dicer1 in HEK293T cells (Figure 3A). Together, the above results substantiated that Dicer1 is a client protein for HSP90 and Aha1.

### Aha1 modulates Dicer1 protein level in HEK293T cells through mechanisms that are dependent on or independent of HSP90

We next explored how HSP90–Aha1 interaction influences the expression level of Dicer1. To this end, we overexpressed wild-type Aha1, Aha1-E67K mutant, which is defective in binding with HSP90 (41), and Aha1-Δ20, where the first 20 amino acids required for its intrinsic chaperoning activity were deleted (42,43), in HEK293T cells with the endogenous Aha1 being ablated with CRISPR/Cas9. Our results showed that reconstitution of Aha1-ablated cells with wild-type Aha1 substantially restored the level of Dicer1 protein; complementation with Aha1-E67K could also rescue the decreased level of Dicer1 protein elicited by ablation of endogenous Aha1, albeit to a lower degree than wild-type Aha1. Aha1-Δ20, however, failed to rescue Dicer1 protein level (Figure 3B–E). In addition, our immunoprecipitation results showed that wild-type Aha1, Aha1-E67K and Aha1-Δ20 can interact with Dicer1 (Supplementary Figures S6 and S7). Aha1-E67K, however, displays diminished interaction with HSP90 compared with wild-type Aha1 (Supplementary Figure S6). These results suggest that Dicer1 is a client protein of Aha1.

**Figure 4.**
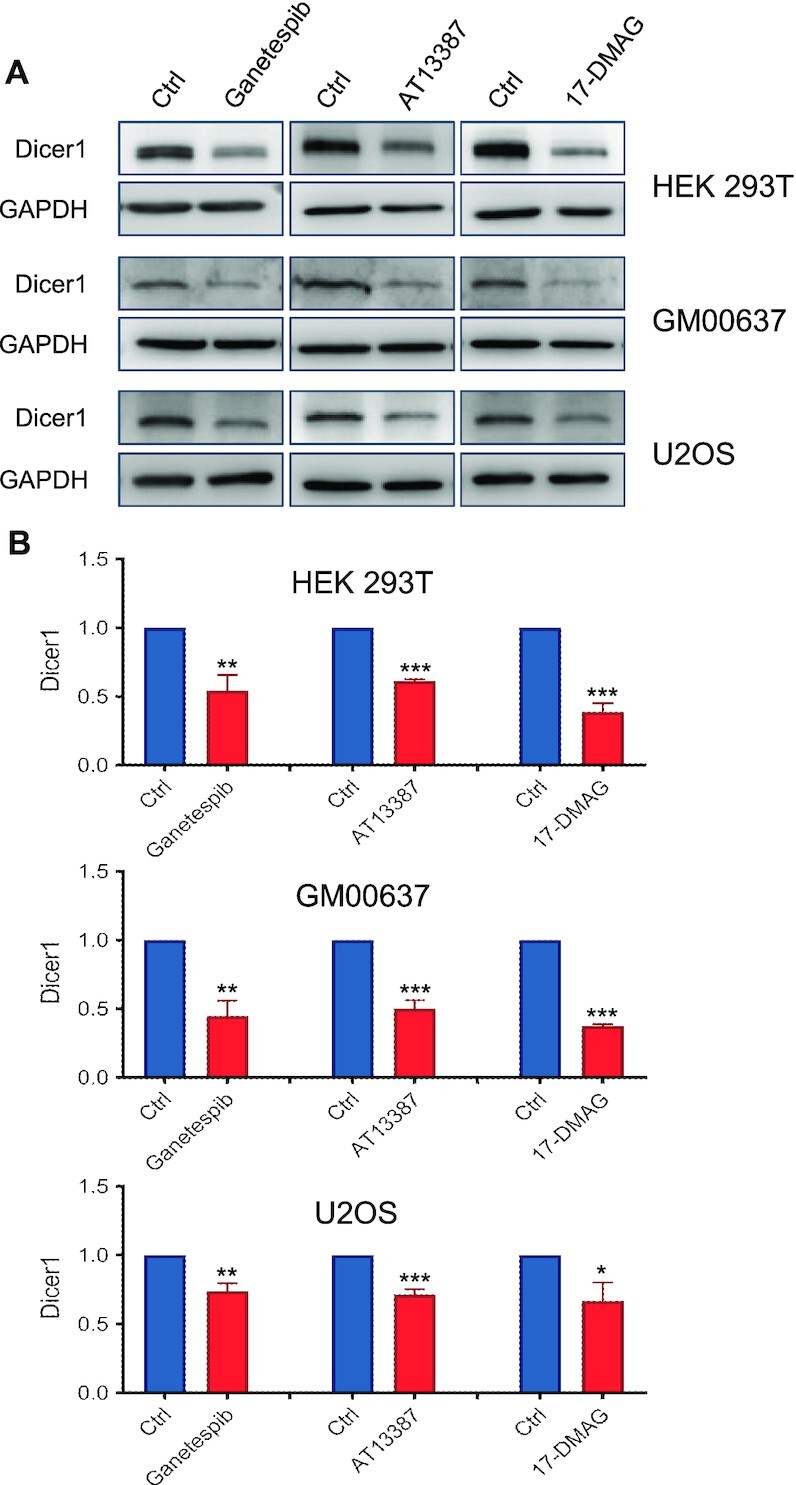
Pharmacological inhibition of HSP90 led to decreased expression of Dicer1 protein in multiple cell lines. (**A**) HEK293T, GM00637 and U2OS cells were treated with HSP90 inhibitors; the cell lysates were employed to monitor the changes in expression level of Dicer1 protein. (**B**) Quantification of Dicer1 protein levels in HEK293T, GM00637 and U2OS cells after treatment with different HSP90 inhibitors. The data were quantified from band intensities using ImageJ and normalized against that of GAPDH, where the values are displayed relative to those observed in mock-treated cells. The data represented the mean ± SD (*n* = 3). The *P* values were calculated using unpaired, two-tailed Student’s *t*-test: *, 0.01 ≤ *P* < 0.05; **, 0.001 ≤ *P* < 0.01; ***, *P* < 0.001.

We also examined the alterations in expression level of Dicer1 in response to treatment with three small-molecule inhibitors of HSP90, i.e. ganetespib, AT13387 and 17-DMAG (also known as alvespimycin) (44). Our results showed that Dicer1 exhibits markedly diminished expression in HEK293T, GM00637 and U2OS cells upon treatment with the three HSP90 inhibitors (Figure 4).

**Figure 5.**
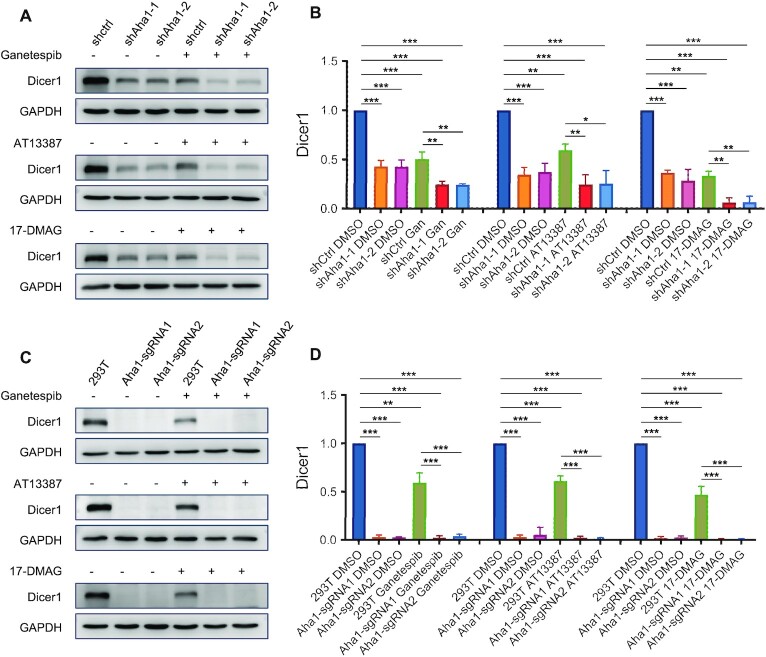
HSP90 and Aha1 act synergistically in regulating Dicer1. (**A**) HEK293T cells with genetic depletion of Aha1 were treated with three HSP90 inhibitors, and cell lysates were used to monitor the alterations in expression levels of Dicer1 protein. (**B**) The changes in Dicer1 protein levels in panel (A). (**C**) HEK293T cells with Aha1 being knocked out with CRISPR using two separate sgRNAs were treated with HSP90 inhibitors, and cell lysates were used to monitor the alterations in expression levels of Dicer1 protein. (**D**) The alterations in Dicer1 protein levels in panel (C). The data were quantified from band intensities using ImageJ and normalized against that of GAPDH, where the values are displayed relative to the level in shControl cells. The data are presented as the mean ± SD (*n* = 3). The *P* values were calculated using unpaired, two-tailed Student’s *t*-test: *, 0.01 ≤ *P* < 0.05; **, 0.001 ≤ *P* < 0.01; ***, *P* < 0.001.

Next, we asked whether the expression level of Dicer1 protein can be further attenuated in cells with simultaneous inhibition of HSP90 and genetic depletion of Aha1. Our results showed that treatment with HSP90 inhibitor, in combination with shRNA-mediated depletion of Aha1, led to more pronounced diminutions in Dicer1 protein level than either treatment alone (Figure 5A and B). Furthermore, genetic ablation of Aha1 results in a much more pronounced decrease of Dicer1, and treatment with HSP90 inhibitors did not further reduce the Dicer1 protein level in the Aha1 knockout background (Figure 5C and D). Together, these results underscore that Aha1 promotes the folding of Dicer1 both through its autonomous chaperone activity and through its role as an HSP90 co-chaperone. This is in agreement with the established functions of Aha1 in serving as an autonomous chaperone to prevent the aggregation of stressed proteins (42).

### Aha1 and HSP90 modulate the maturation of miRNAs

miRNAs, which comprise a large family of ∼21-nt-long RNAs, constitute a key post-transcriptional regulatory mechanism of gene expression (45). Pre-miRNAs are transported into the cytoplasm and processed further by Dicer proteins (37,38) to produce a double-stranded species consisting of a passenger strand that is degraded (45) and an miRNA guide strand, which is delivered to the RNA-induced silencing complex (RISC) to mediate the cleavage and/or translational suppression of its target mRNA(s) (46).

**Figure 6.**
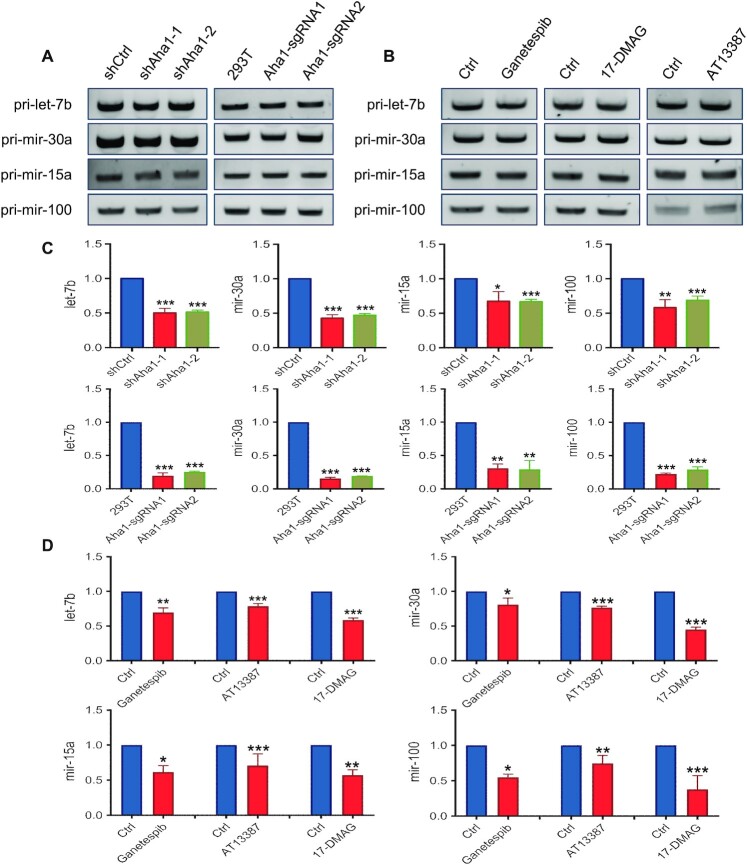
HSP90–Aha1 pathway regulates miRNA maturation. RT-PCR results showed that (**A**) shRNA knockdown or CRISPR knockout of Aha1 or (**B**) treatment with HSP90 inhibitors did not affect the levels of primary transcripts of let-7b, mir-30a, mir-15a and mir-100 in HEK293T cells. (**C**) RT-qPCR results showed that the mature miRNA levels of let-7b, mir- 30a, mir-15a and mir-100 exhibited significant decreases after genetic depletion of Aha1 by using CRISPR or shRNA in HEK293T cells. (**D**) The mature miRNA levels of let-7b, mir-30a, mir-15a and mir-100 in HEK293T cells after treatment of HSP90 inhibitors. The data were normalized to the mRNA level of *GAPDH* gene. The quantification data represent the mean ± SD of results from three independent experiments. The *P* values were calculated based on unpaired, two-tailed Student’s *t*-test: *, 0.01 ≤ *P* < 0.05; **, 0.001 ≤ *P* < 0.01; ***, *P* < 0.001.

The above results showed that Dicer1 is a client protein of HSP90 and Aha1. Hence, we next investigated whether HSP90 and Aha1 are involved in the maturation of let-7b, mir-30a, mir-15a and mir-100, which are highly abundant in HEK293T cells (47). Our results showed that genetic depletion of Aha1 by using CRISPR or shRNA led to decreased levels of mature let-7b, mir-30a, mir-15a and mir-100 in HEK293T cells, with more pronounced decreases being observed for the CRISPR-engineered cells than the shRNA-treated cells (Figure 6). This is in line with more marked diminutions in the levels of Dicer1 in Aha1 CRISPR knockout cells than shRNA knockdown cells (Figures 2, 3 and 5). Genetic depletion of Aha1 with CRISPR or shRNA, however, did not affect the levels of their primary miRNAs (Figure 6 and Supplementary Figure S8). Likewise, suppression of HSP90 activity with the three small-molecule inhibitors attenuated the levels of mature let-7b, mir-30a, mir-15a and mir-100, and we again did not observe any appreciable alterations in the levels of their primary miRNAs (Figure 6 and Supplementary Figure S8). These results are in agreement with the aforementioned observations made for Dicer1 protein, and demonstrated that HSP90 and Aha1 modulate miRNA maturation through enhancing the maturation and folding of Dicer1 protein.

## DISCUSSION

HSP90 is a molecular chaperone that is conserved from bacteria to humans, and it facilitates the maturation and folding of client proteins, which are involved in many different cellular pathways (4). During client processing, a large number of co-chaperones interact with HSP90 to regulate the ATPase-associated conformational changes of the HSP90 dimer. In this vein, CDC37 is a well-characterized client-specific co-chaperone, and protein kinases are its best characterized clients (5,48). Aha1 is a potent activator of HSP90’s ATPase function, and it is known to modulate proteins involved in cystic fibrosis and Alzheimer’s disease (17,18).

By employing APEX labeling, together with label-free quantification, we uncovered a number of client proteins of Aha1 in HEK293T cells. Our results revealed that, upon genetic depletion of Aha1, 31 proteins exhibit diminished presence in the proximity proteome of HSP90 (Figure 1 and Supplementary Table S1). We further validated the proteomic data for Dicer1 by streptavidin affinity pulldown followed by western blot analysis (Figure 2).

We found that knockdown of Aha1 led to decreased expression of Dicer 1 (Figure 2 and Supplementary Figure S1), and immunoprecipitation followed by western blot analysis revealed the interaction between endogenous HSP90 and Dicer1, and between Aha1 and Dicer1 (Figure 2 and Supplementary Figure S5). Furthermore, we observed preferential binding of HSP90 and Aha1 with newly synthesized Dicer1, and the expression level of Dicer1 can be reduced upon genetic depletion by Aha1 and pharmacological inhibition of HSP90, with more pronounced effect being observed for functional suppression of both Aha1 and HSP90 than either suppression alone (Figures 3–5). Rescue experiments showed that complementation with wild-type Aha1 could markedly restore the level of Dicer1 elicited by genetic ablation of Aha1. The HSP90-binding-defective Aha1-E67K could also restore Dicer1 protein level, but to a lesser degree than its wild-type counterpart; however, Aha1-Δ20, which abolishes its chaperone activity, failed to rescue it (Figure 3). These results revealed that Aha1 can modulate the protein level of Dicer1 by pathways that are dependent on or independent of HSP90. Our findings are consistent with previous finding that Aha1 can function as an HSP90-independent, autonomous chaperone. In this vein, Tripathi *et al.* (42) observed that the majority of Aha1 exists in cells as a self-contained protein independent of high-molecular-weight HSP90 protein complexes and Aha1 itself can prevent the aggregation of denatured rhodanese and luciferase. In addition, Mollapour *et al.* (16) showed that diminished HSP90–Aha1 interaction associated with reduced ATPase stimulation bears little effect on the activation of some client proteins, suggesting an additional role of Aha1 beyond its function as a potent stimulator of the HSP90’s ATPase activity.

We also uncovered that mature miRNA levels were decreased upon genetic depletion of Aha1 or pharmacological inhibition of HSP90 (Figure 6), which is in keeping with Dicer1’s functions in miRNA maturation (37,38). In this regard, it was observed that HSP90 interacts with Argonaute 2 (Ago2), which is the catalytic engine of the RISC, and this interaction modifies the conformation of Ago2 protein, thereby enabling it to receive double-stranded RNA from the RISC-loading complex (49,50), which is known to contain Dicer (51). Moreover, HSP90 activity is important for stable interaction between Argonaute proteins and Dicer (52). Hence, these previous observations, together with our findings, support that HSP90 plays a pivotal role in multiple steps of miRNA process (i.e. maturation of miRNAs and their subsequent loading to RISC) through regulating both Ago2 and Dicer1. In this context, it is of note that our APEX labeling data did not reveal the presence of Ago2 in the proximity proteome of HSP90. The exact reason is unclear, although this could be potentially attributed to the transient and/or weak interaction between the two proteins. In addition, we fused APEX2 to the C-terminus of HSP90, and the interaction with Ago2 may involve region(s) distal from the C-terminal domain of HSP90; hence, it will be interesting to explore whether fusion of APEX2 to the N-terminus of HSP90 could allow for the identification of Ago2 in the proximity proteome of HSP90.

Viewing that ∼30% of human genes are subjected to post-transcriptional regulation via miRNA-dependent mechanisms (10), our work also unveiled the important roles of HSP90 and Aha1 in modulating gene expression. In this vein, our proteomic data also revealed elevated presence of a number of proteins in the proximity proteome of HSP90 upon genetic depletion of Aha1 (Figure 1 and Supplementary Table S1). While the exact mechanisms remain unclear, we reason that attenuated maturation of miRNAs and the ensuing increased translation of their target mRNAs may contribute, in part, to the increased existence of these proteins in close proximity of HSP90.

Together, we uncovered, for the first time, Dicer1 as a client protein of Aha1. Reduced expression of Dicer1 is known to be associated with various cancers (53), and miRNAs may exert oncogenic or tumor-suppressing functions under certain conditions (54). Therefore, our discovery about the regulatory roles of HSP90 and Aha1 in Dicer1 protein expression and miRNA maturation may provide an important mechanistic basis for developing therapeutic approaches toward cancer treatment. It will be important to examine, in the future, the domains of Dicer1 protein that are involved in interacting with Aha1 and HSP90. Moreover, it can be envisaged that the methods reported here can be employed to uncover client proteins of other co-chaperones of HSP90.

## DATA AVAILABILITY

Excel files containing the processed MS data are provided in the Supplementary Data. The raw files and the MaxQuant analyses were deposited into ProteomeXchange (PXD-028980).

## Supplementary Material

gkac528_Supplemental_File
