# Supplementary material for: HSP90 and Aha1 modulate microRNA maturation through promoting the folding of Dicer1
Source: Nucleic Acids Res. 2022 Jun 23;50(12):6990–7001. doi: 10.1093/nar/gkac528 (PMC9262616; doi:10.1093/nar/gkac528)
Supplement: gkac528_Supplemental_File [file gkac528_supplemental_file.pdf]

Supplementary Materials for

"HSP90 and Aha1 Modulate MicroRNA Maturation through Promoting the Folding of Dicer1"

Xiaochuan Liu, Yen-Yu Yang and Yinsheng Wang*

Department of Chemistry, University of California, Riverside, Riverside, CA 92502, USA.

*To whom correspondence should be addressed. Email: yinsheng@ucr.edu.

Supplementary Table 1. List of proteins quantified in the APEX-based HSP90 proximity proteome analysis in HEK293T cells with genetic depletion of Aha1. The LFQ intensity of protein from Aha1 knockdown background with two separate sequences of shRNAs (shA1, shA2) was normalized against that of shControl (shCtrl). Proteins identified only in shControl were labeled as Ctrl only. N/A represents missing data.

Gene names	shA1/shCtrl-1	shA1/shCtrl-2	shA1/shCtrl-3	shA2/shCtrl-1	shA2/shCtrl-2	shA2/shCtrl-3
AHSA1	Ctrl only	Ctrl only	Ctrl only	Ctrl only	Ctrl only	Ctrl only
DICER1	Ctrl only	Ctrl only	Ctrl only	Ctrl only	Ctrl only	Ctrl only
SULT1A1	Ctrl only	Ctrl only	Ctrl only	Ctrl only	Ctrl only	Ctrl only
TMED10	0.387	0.714	0.501	0.53	0.519	0.531
TMED9	0.534	0.54	0.355	Ctrl only	0.588	0.428
TMED7	0.281	Ctrl only	Ctrl only	0.372	0.41	0.324
SLC7A1	Ctrl only	0.453	0.567	Ctrl only	Ctrl only	Ctrl only
SLC3A2	0.619	0.613	0.724	0.765	0.56	0.496
SLC12A2	Ctrl only	Ctrl only	Ctrl only	1.306	Ctrl only	Ctrl only
SLC25A24	Ctrl only	Ctrl only	N/A	Ctrl only	Ctrl only	N/A
ABCC1	N/A	Ctrl only	Ctrl only	N/A	Ctrl only	Ctrl only
ABCB10	0.459	0.668	Ctrl only	1.677	Ctrl only	Ctrl only
WARS	Ctrl only	Ctrl only	Ctrl only	Ctrl only	Ctrl only	Ctrl only
ACTR2	Ctrl only	Ctrl only	Ctrl only	Ctrl only	Ctrl only	Ctrl only
CLPX	Ctrl only	0.739	0.625	Ctrl only	Ctrl only	0.721
ETHE1	Ctrl only	Ctrl only	0.902	Ctrl only	1.223	Ctrl only
PGK1	Ctrl only	N/A	Ctrl only	Ctrl only	N/A	Ctrl only
PCCA	0.614	0.593	0.517	1.009	0.596	0.648
AK4	0.346	0.376	0.417	0.547	0.303	0.308
CPOX	Ctrl only	Ctrl only	1.076	Ctrl only	Ctrl only	Ctrl only
EIF2S3	0.656	0.597	0.578	0.541	0.549	0.692
RAB11A	Ctrl only	0.62	0.747	Ctrl only	0.525	0.417
TUBB3	Ctrl only	Ctrl only	Ctrl only	0.647	0.454	Ctrl only
TUBB2A	0.819	Ctrl only	0.655	Ctrl only	Ctrl only	Ctrl only
PCK2	0.363	0.34	0.386	0.425	0.296	0.358
AMOT	Ctrl only	Ctrl only	N/A	0.563	Ctrl only	N/A
STX12	0.974	Ctrl only	Ctrl only	1.309	0.586	Ctrl only
GCN1L1	N/A	Ctrl only	Ctrl only	N/A	Ctrl only	Ctrl only
EIF3K	Ctrl only	N/A	Ctrl only	Ctrl only	N/A	Ctrl only
SEL1L	Ctrl only	0.629	Ctrl only	Ctrl only	Ctrl only	Ctrl only
COQ6	Ctrl only	1.028	Ctrl only	1.009	Ctrl only	Ctrl only

Supplementary Table 2. Sequences for shRNA and overexpression plasmids primers.

Description	Primer
shAha1-1 Forward	5'-CCGGCGGTACTACTTTGAGGGCATTCTCGAGAATGCCCTCAAAGTAGTACCGTTTTTG-3'
shAha1-1 Reverse	5'-AATTCAAAAACGGTACTACTTTGAGGGCATTCTCGAGAATGCCCTCAAAGTAGTACCG-3'
shAha1-2 Forward	5'-CCGGCCCTGAGAAACATATTGTGATCTCGAGATCACAATATGTTTCTCAGGGTTTTTG-3'
shAha1-2 Reverse	5'-AATTCAAAAACCCTGAGAAACATATTGTGATCTCGAGATCACAATATGTTTCTCAGGG-3'
Aha1-Flag Forward	5'-CGGGATCCATGGCCAAGTGGGGTGAGGGAGAC-3'
Aha1-Flag Reverse	5'-CGGAATTCCTAAAATAAGCGTGCGCCATAGCCAAAGGT-3'
Aha1 E67K-Flag Forward	5'-TAATGGATGCCTTTCCATCAAGCTTACTCACTTCCG-3'
Aha1 E67K-Flag Reverse	5'-CGGAAGTGAGTAAGCTTGATGGAAAGGCATCCATTA-3'
Aha1 deletion 20 Forward	5'-AAAGGATCCAACGTCAACAACTGGCACTGGACGGA-3'
Aha1 deletion 20 Reverse	5'-AAAGAATTCCTAAAATAAGCGTGCGCCATAGCCAAAG -3'
Aha1-EGFP Forward	5'-AAAGCTAGCATGGCCAAGTGGGGTGAGGGAGAC-3'
Aha1-EGFP Reverse	5'-AAAACCGGTTTAAATAAGCGTGCGCCATAGCCAAAGGT-3'
Aha1 E67K-EGFP Forward	5'-TAATGGATGCCTTTCCATCAAGCTTACTCACTTCCG-3'
Aha1 E67K-EGFP Reverse	5'-CGGAAGTGAGTAAGCTTGATGGAAAGGCATCCATTA-3'
HSP90 Forward	5'-ATAAGAATGCGGCCGCATGCCTGAGGAAGTGCACCATGGAGAGGA-3'
HSP90 Reverse	5'-CTAGCTAGCCTAATCGACTTCTTCCATGCGAGACGCATC-3'
Aha1-sgRNA1 top	5'-CACCGAAACTTCTAAGAGAAGCAAT -3'
Aha1-sgRNA1 bottom	5'-AAACATTGCTTCTCTTAGAAGTTTC -3'
Aha1-sgRNA2 top	5'-CACCGGAGAGTCAGTAGACCCAGTG -3'
Aha1-sgRNA2 bottom	5'-AAACCACTGGGTCTACTGACTCTCC -3'

Supplementary Table 3. Primer sequences for miRNA RT-qPCR

Description	Primer
5′ tag-oligo dT_{15}	5'-CAGGTCCAGTTTTTTTTTTTTTTT -3'
let-7b Forward	5'-GCAGTGAGGTAGTAGGTTGTGT -3'
let-7b Reverse	5'-CAGGTCCAGTTTTTTTTTTTTTTTAACCACAC -3'
let-7b synthetic template	5'-CAGGTCCAGTTTTTTTTTTTTTTTAACCACACAACCTACTACCTCA -3'
mir-30a Forward	5'-CGCAGTGTAAACATCCTCGACTG -3'
mir-30a Reverse	5'-CAGGTCCAGTTTTTTTTTTTTTTTCTTCCAG -3'
mir-30a synthetic template	5'-CAGGTCCAGTTTTTTTTTTTTTTTCTTCCAGTCGAGGATGTTTACA -3'
mir-15a Forward	5'-CGCAGTAGCAGCACATAATGGTT -3'
mir-15a Reverse	5'-CAGGTCCAGTTTTTTTTTTTTTTTCACAAAC -3'
mir-15a synthetic template	5'-CAGGTCCAGTTTTTTTTTTTTTTTCACAAACCATTATGTGCTGCTA -3'
mir-100 Forward	5'-GCAGAACCCGTAGATCCGAACT -3'
mir-100 Reverse	5'-CAGGTCCAGTTTTTTTTTTTTTTTCACAAGTT -3'
mir-100 synthetic template	5'-CAGGTCCAGTTTTTTTTTTTTTTTCACAAGTTCGGATCTACGGGTT -3'

Supplementary Table 4. Primer sequences for precursor miRNA RT-PCR

Description	Primer
pri-let-7b F	5'-CAGAGAGCCAGGGACTTCCC -3'
pri-let-7b R	5'-CTAGCTCCCAGATGCCCACC -3'
pri-mir 30a F	5'-GCTGTTTGAATGAGGCTTCAG -3'
pri-mir 30a R	5'-CACAGAGCACCTCCTCAATG -3'
pri-mir 15a F	5'-AACCTATAGCACTGTGCTGGGCA -3'
pri-mir 15a R	5'-AACGCCAATATTTACGTGCTGCTAA -3'
pri-mir 100 F	5'-GCTTTTGTGAGAGTGGGACGAAGT -3'
pri-mir 100 R	5'-TCATTGAAGAACCTCATTCATTTCAGGACAA -3'

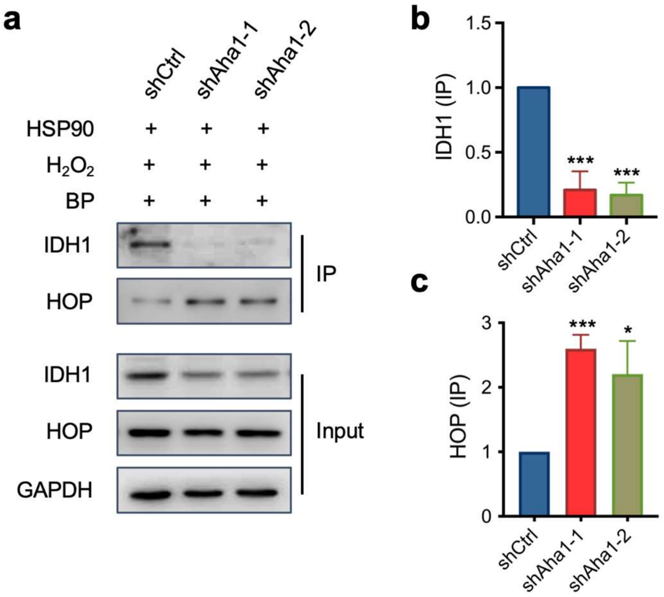

Supplementary Figure 1. Genetic depletion of Aha1 led to diminished levels of IDH1, and increased levels of HOP in the proximity proteome of HSP90. (a) HEK293T cells treated with shAha1 and shControl were transfected with HSP90-APEX plasmid for 24 h, treated with biotin phenol for 30 min and H_2O_2 for 1 min, followed by cell lysis, streptavidin affinity purification, and the whole cell lysate and pull-down lysate were used for Western blot analysis. (b-c) The alteration in the levels of IDH1 and HOP in the proximity proteome of HSP90 based on IP panel of (a). IDH1 and HOP levels in (b-c) were quantified from band intensities using ImageJ and were displayed relative to the level in HEK293T shControl cells. The data represented the mean ± S.D. (n = 3). The *p* values were calculated using unpaired, two-tailed Student's *t*-test: *, $0.01 \leq p < 0.05$; **, $0.001 \leq p < 0.01$; ***, $p < 0.001$.

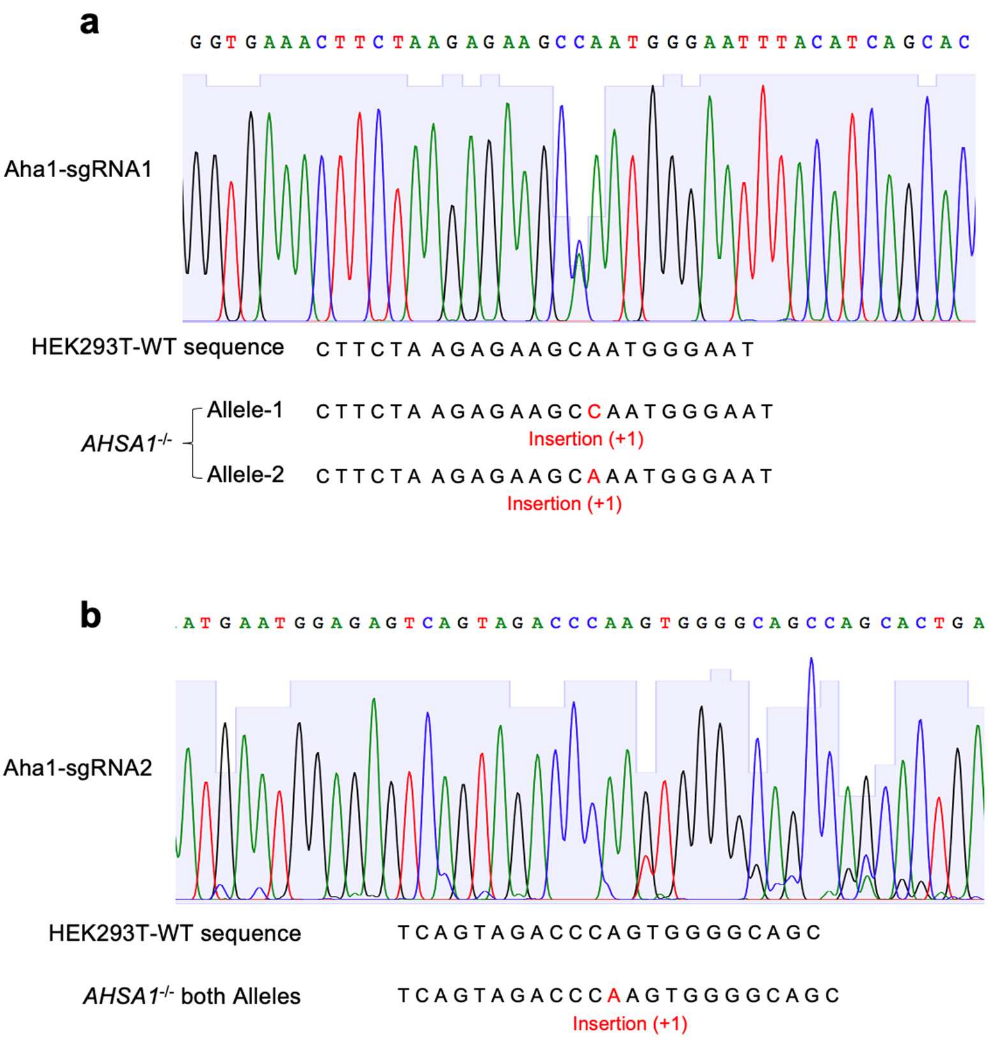

Supplementary Figure 2. Representative Sanger sequencing results showing CRISPR-induced indel mutations of *AHSA1* gene in HEK293T cells with two separate sequences of sgRNAs targeting exon4 (a) and exon5 (b) of Aha1.

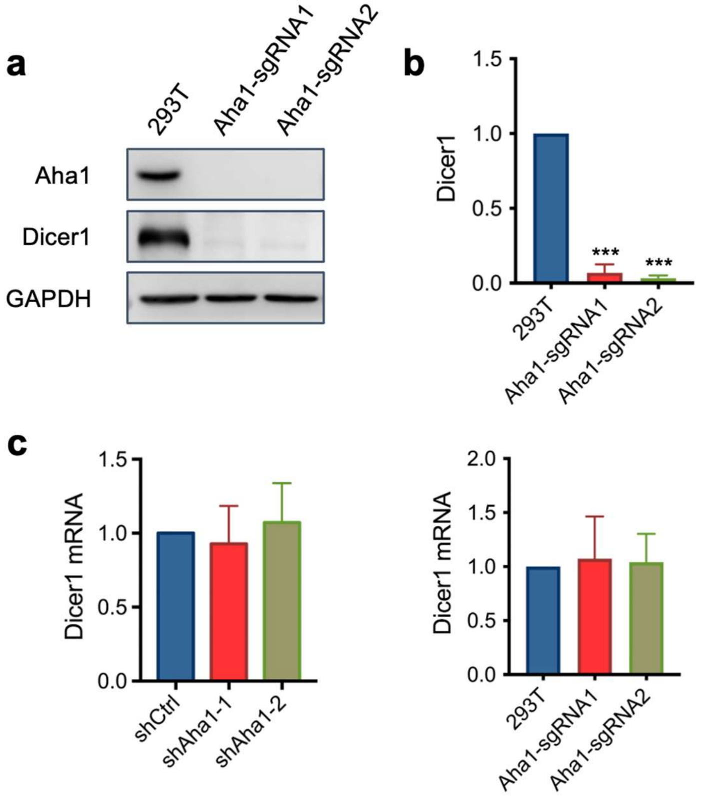

Supplementary Figure 3. CRISPR-mediated ablation of Aha1 in HEK293T cells led to decreased protein levels of Dicer1, but not its mRNA levels. (a) Western blot analysis showed the expression level of Dicer1 protein after CRISPR-mediated knockout of Aha1 in HEK293T cells. (b) Quantification of Dicer1 protein level affected by knockout of Aha1 in HEK293T cells. (c) Quantification of Dicer1 mRNA level affected by shRNA-mediated knockdown and CRISPR-mediated knockout of Aha1 in HEK293T cells. The data were quantified from band intensities using ImageJ and normalized against that of GAPDH, where the values are displayed relative to those observed in shControl cells. The data represented the mean ± S.D. (n = 3). The p values were calculated using unpaired, two-tailed Student's t-test: ***, $p < 0.001$.

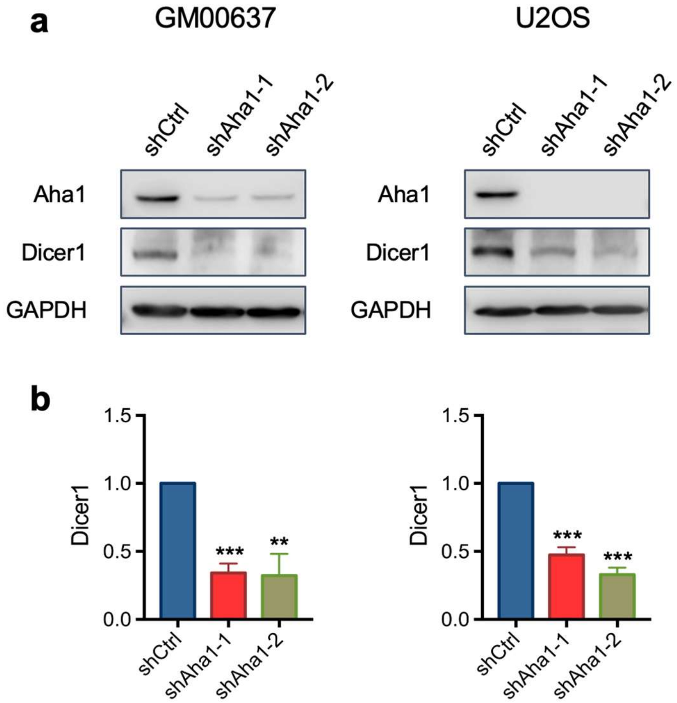

Supplementary Figure 4. Dicer1 level was decreased after genetic depletion of Aha1 in different cell lines. (a) Western blot analysis showed the expression level of Dicer1 protein after knockdown of Aha1 in GM00637 and U2OS cells. (b) Quantification of Dicer1 level affected by knockdown of Aha1 in GM00637 and U2OS cells. The data were quantified from band intensities using ImageJ and normalized against that of GAPDH, where the values are displayed relative to those observed in shControl cells. The data represented the mean ± S.D. (n = 3). The *p* values were calculated using unpaired, two-tailed Student's *t*-test: **, $0.001 \le p < 0.01$; ***, $p < 0.001$.

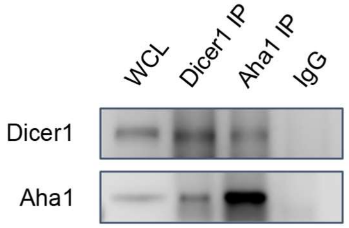

Supplementary Figure 5. Endogenous Dicer1 interacts with endogenous Aha1. The lysates of HEK293T cells were immunoprecipitated with antibodies against endogenous Dicer1 and Aha1, and the immunoprecipitates were employed to monitor the levels of Aha1 and Dicer1 proteins by Western blot.

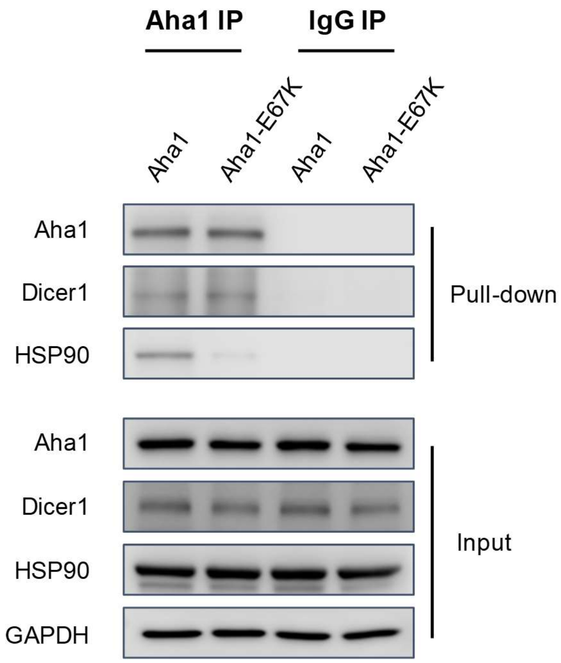

Supplementary Figure 6. Aha1-E67K displays weaker interaction with HSP90 than wild-type Aha1; the two variants of Aha1, however, exhibit similar interaction with Dicer1. HEK293T *AHSA1*$^{-/-}$ cells were transfected with plasmids for expressing wild-type Aha1 and Aha1-E67K for 24 h, followed by cell lysis, pull-down using anti-Aha1 or control IgG, and the whole-cell lysate and pull-down lysate were used for Western blot analysis.

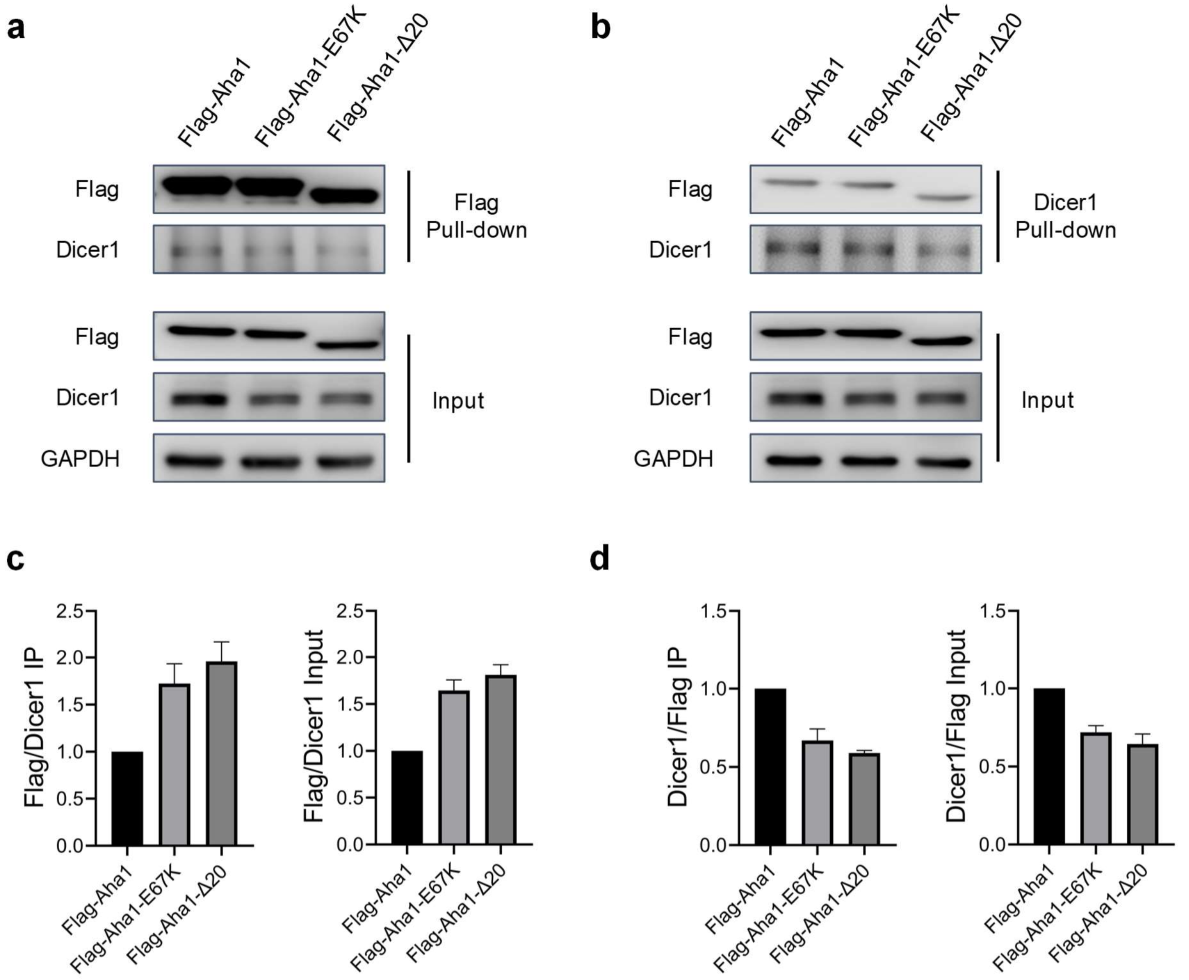

Supplementary Figure 7. Aha1-Δ20 interacts with Dicer1. (a) HEK293T cells were transfected with plasmids for expressing Flag-Aha1, Flag-Aha1-E67K and Flag-Aha1-Δ20 for 24 h, followed by cell lysis, and pull-down using anti-Flag. (b) Reciprocal pull-down using anti-Dicer1 antibody of the same cell lysates as in (a), and the whole-cell lysate and pull-down lysate were used for Western blot analysis. (c-d) Quantification of Flag/Dicer1 and Dicer1/Flag ratios based on IP panel and Input panel of (a) and (b), respectively. The data represented the mean ± S.D. (n = 2).

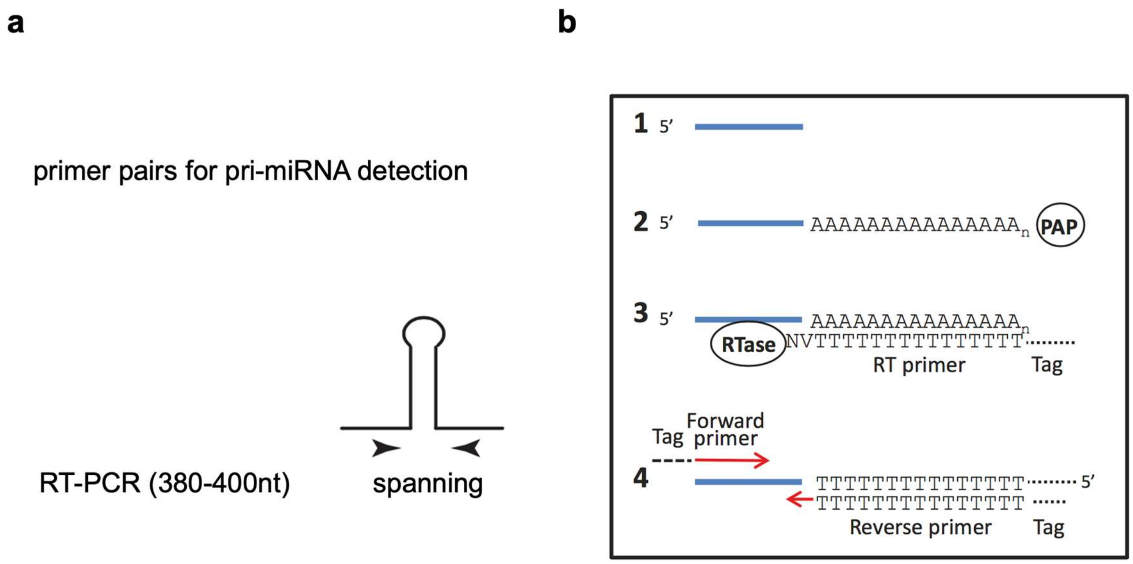

Supplementary Figure 8. Primer design for primary miRNA detection and qPCR work flow of mature miRNA measurement. (a) Primer pairs for pri-miRNA detection.[1] (b) A schematic diagram illustrating the procedures of RT-qPCR for quantifying specific mature miRNA.[2]

References

1. Yeom, K. H.; Mitchell, S.; Linares, A. J.; Zheng, S.; Lin, C. H.; Wang, X. J.; Hoffmann, A.; Black, D. L., Polypyrimidine tract-binding protein blocks miRNA-124 biogenesis to enforce its neuronal-specific expression in the mouse. *Proc. Natl. Acad. Sci. USA* **2018,** *115*, E11061-E11070.

2. Balcells, I.; Cirera, S.; Busk, P. K., Specific and sensitive quantitative RT-PCR of miRNAs with DNA primers. *BMC. Biotechnol.* **2011,** *11*, 70.
